# A Splenectomy, with Remarks

**Published:** 1902-05

**Authors:** Jno. G. Earnest

**Affiliations:** Atlanta, Ga.


### A SPLENECTOMY, WITH REMARKS.

BY JNO. G. EARNEST, M.D.,
ATLANTA, GA.

The function of the spleen is one of the unsolved mysteries of science. For a long time it was supposed to be only a reservoir for the blood when driven from the surface, or dammed up by obstruction in some part of the venous circulation. The fact that in disease of the lungs, the heart, or the liver, there is no corresponding enlargement of the spleen, while in conditions of profound anemia and deficient blood supply the spleen is frequently enormously enlarged disproves that theory. Of late years it has been claimed that it plays an important part in digestion—a theory that seems to be extremely problematical, in view of the fact that many individuals have had the spleen removed and have suffered no inconvenience from it after the economy has had time to adjust itself to the new state of affairs. Richard Douglass in *Journal of the American Medical Association*, for April 25, '96, reports one hundred and twenty-seven splenectomies that he had collected and classified as follows: 36 cases of leukemia with 5 recoveries (16 deaths from secondary hemorrhage and 5 from shock), 40 malarial cases with 24 recoveries, 59 cases of simple hypertrophy with 34 recoveries (9 deaths from hemorrhage), 5 cases of sarcoma with 2 recoveries, 8 other cases (6 of which were for cysts) with 5 recoveries. In 4 of these cases the blood was examined before and after operation. In two the red corpuscles were increased, in the other two they were diminished. In a series of experiments conducted by Blumerich and Jacoby (Berl. Klin. Woch. May 24, '97) in which the spleen was extirpated in 200 guinea pigs, it was found that there was marked increase of leucocytes in the blood. This experience has been confirmed by operations on the human subject. The animals in the above series were afterwards injected with various toxines and germs. In the cases infected with diphtheria, cholera and pyocyaneus bacilli the animals seemed to have acquired increased power of resistance which was attributed to the destructive forces of the increased number of leucocytes. Recent experience shows that splenectomy is followed by marked decrease of hemogoblin and red blood corpuscles and a corresponding increase in white corpuscles. In successful cases the equilibrium is restored in from fifteen to twenty days. The operation is usually followed by considerable systemic disturbance, the prominent features of which are anemia, nausea, griping pains in bowels, diarrhea, rise of temperature, very rapid pulse, great thirst, dizziness, stupor, pains in the limbs and enlargement of the lymphatics. Recovery is usually slow, but is eventually complete.

It is not within the scope of this paper to take up the diseases of the spleen or even to allude to the great variety of shapes, size and position of diseased spleens, but simply to report a case recently operated upon which illustrates many of the points above mentioned, and, so far as my acquaintance with the subject goes, is unique and important from a diagnostic standpoint.

The patient, age 33, was reared in a malarious region in Southern Georgia, and from her earliest recollection was subject to frequent attacks of malarial fever. When she was twenty-five years old she married. Two years ago she became pregnant, but miscarried. After the miscarriage her health declined and she had painful, irregular menstruation. In November, 1900, she noticed a swelling about the lower margin of the ribs on the left side. This tumor caused pain by pressing against the ribs. Gradually the mass enlarged and sunk lower in the abdomen until it rested in the pelvis, pressing against the uterus and bladder, causing constant pain when on her feet and a continuous desire to micturate. This state of affairs finally compelled her to take her bed. She was sent to a sanitorium, where she remained for several months, returning home without relief. After remaining some months at home under treatment of her family physician and growing rather worse than better, she, by his advice, came to Atlanta. On December 15th, 1901, I was asked by Dr. K. H. Boland to see her, and found her confined to bed, complaining of pain in the ovarian region and bladder. Her digestion was poor and she was profoundly anemic. I found a mass filling the pelvis and standing out above the pelvic brim on each side. The mass was firmly fixed and could not be moved in any position of the patient. The outline could be traced in the bottom of the pelvis underlying the fundus uteri and crowding the cervix up against the pubis. Operation was advised and accepted. After the usual preparation, on December 19, assisted by Drs. K. H. Boland, Haywood Hansell and N. W. Earnest, the abdomen was opened under ether. The mass was found to be the displaced spleen about 22 centimetres in length, crowded down into the pelvis with the convex surface at the bottom and the two ends brought up in such a manner as to give it a crescentic shape. A deep transverse sulcus across the lower third gave the lower portion the appearance of a distinct lobe. On top of the mass rested the uterus. On the left side the ovary and fimbriated extremity were united to the spleen by light adhesions. The spleen was adherent to the abdominal walls, but peeled out without difficulty. The organ was lifted out and the vessels secured by a stout double ligature and the mass cut off, care being taken to give ample length to the pedicle to prevent the slipping of the ligature. The abdomen was closed with silkworm gut. She reacted promptly and did not suffer seriously from ether nausea. On the second day her fever rose to 101 and she had some nausea. From this on her fever gradually rose until the seventh day, when it reached 104 in the evening. In the meantime she had nausea with occasional vomiting and diarrhea with severe griping. Suspecting that this might be partly due to malaria, she was freely dosed with quinine, and in three or four days the symptoms began to decline, so that at the end of the second week the fever, the vomiting and the diarrhea were gone and she began to have a little appetite. Her digestive powers, however, were greatly impaired, and she could take only the most simple food in small quantities. When she left for home the middle of February, she was still weak, but able to walk about and was comfortable. When last heard from, a month after she went home, her digestion was much better and she was still gaining in strength and weight.

